# “Dissolving Cartesian dualism”: using a novel student-lead podcast to explore the relationship between neurological and psychiatric illness

**DOI:** 10.1192/j.eurpsy.2022.1163

**Published:** 2022-09-01

**Authors:** S. James

**Affiliations:** University of Bristol, Medical School, Bristol, United Kingdom

**Keywords:** medical students, podcast, neurology, undergraduate

## Abstract

**Introduction:**

Psychiatric conditions can be both a symptom and a consequence of physical disease. Although understanding of this is important for health care delivery, coverage of the relationship between physical and psychiatric illness in undergraduate medical education is sparse. This relationship is particularly pertinent in neurological disorders, where psychiatric symptomatology is commonplace. As part of a student project, the author was tasked with developing accessible teaching materials to increase interest and understanding among medical students, using podcasts.

**Objectives:**

The aim was to develop podcasts which explored the relationship between psychiatric and neurological illness, for use in undergraduate psychiatric training.

**Methods:**

Literature reviews were performed on podcasting in medical education to identify the optimal methods of production to maximise educational value, and on topics covered in podcasts to inform the interview questions. Experts in the relevant areas were contacted for recorded interviews, later used to create podcasts.

**Results:**

Four interviews were conducted between the author and consultant neurologists specialising in the specific neurological condition. A podcast was produced for each of the following topics: depression in multiple sclerosis, frontotemporal dementia in motor neurone disease, Lewy body dementia, and dissociative seizures.

**Conclusions:**

Psychiatric and physical illness are often intertwined. As the prevalence of psychiatric illness rises, it is becoming increasingly important that this connection is recognised, in order to improve patient experiences and outcomes. Novel teaching modalities, such as podcasts, can provide additional ways to support medical education on this important topic.

**Disclosure:**

No significant relationships.

